# Raf kinase inhibitor protein inhibits cholangiocarcinoma cell metastasis by downregulating matrix metalloproteinase 9 and upregulating tissue inhibitor of metalloproteinase 4 expression

**DOI:** 10.3892/ol.2014.2637

**Published:** 2014-10-24

**Authors:** JUNJI MA, JUNLI SHI, DONGQIANG ZHAO, LIJUAN CHENG, WENBIN WANG, FANGFANG LI, XIAOYU JIANG, HUIQING JIANG

**Affiliations:** 1 Department of Gastroenterology, The Second Hospital of Hebei Medical University, Hebei Key Laboratory of Gastroenterology, Hebei Institute of Gastroenterology, Shijiazhuang, Hebei 050000, P.R. China; 2 Department of Hepatobiliary Surgery, The Second Hospital of Hebei Medical University, Shijiazhuang, Hebei 050000, P.R. China; 3 Department of Biochemistry and Molecular Biology, Basic Medical College of Hebei Medical University, Shijiazhuang, Hebei 050017, P.R. China; 4 Department of Anatomy and Cell Biology, Wayne State University School of Medicine, Detroit, MI 48201, USA

**Keywords:** cholangiocarcinoma, Raf kinase inhibitor protein, invasion, migration

## Abstract

Cholangiocarcinoma cells originate in the biliary epithelium. The cells easily metastasize and cause relapse. The effect of Raf kinase inhibitor protein (RKIP) on the biological behavior of cholangiocarcinoma cells is not yet clear. In the present study, RKIP and cytokeratin 19 expression was detected in the extrahepatic tissues of cholangiocarcinoma patients by immunohistochemistry. RKIP small interfering (si)RNA or an RKIP-overexpressing adenoviral vector were used to infect the human cholangiocarcinoma RBE cell line. RKIP protein or gene expression was analyzed by western blotting or reverse transcription-quantitative polymerase chain reaction (RT-qPCR), respectively. The cells were assayed for proliferation, apoptosis, invasion and migration. Matrix metalloproteinase 9 (MMP-9) and tissue inhibitor of metalloproteinase 4 (TIMP-4) mRNA was assayed by RT-qPCR. RKIP expression was reduced in the extrahepatic cholangiocarcinoma tumor compared with the adjacent uninvolved peritumoral tissues. The current study revealed that RKIP expression was positively correlated with cell differentiation, but negatively correlated with lymph node or distant metastasis (P<0.05). RKIP siRNA treatment promoted RBE cell invasion, but RKIP overexpression prevented cell invasion. In the pDC316-siRNA recombinant vector group, the cells migrated more quickly compared with the siRNA-negative control group, and in the RKIP-expressing adenoviral vector group, the cells migrated more slowly compared with the adenoviral negative control group. RKIP inhibited the invasive and metastatic ability of the cholangiocarcinoma cell line, RBE, by downregulating MMP-9 and upregulating TIMP-4 mRNA expression. RKIP is negatively associated with cholangiocarcinoma distant metastasis and prevents cholangiocarcinoma cell metastasis through downregulating MMP-9 expression and upregulating TIMP-4 expression.

## Introduction

Cholangiocarcinoma is a malignant tumor that originates in the intra- and extrahepatic biliary epithelium and is commonly found in the elderly, being more frequent in males than females. In the early stage of neoplastic transformation, cholangiocarcinoma lacks specific clinical manifestations. Certain common symptoms, including jaundice, usually do not present until late in the course of the disease, at which time they are relatively resistant to chemotherapeutic agents. As such, these symptoms are difficult to treat and exhibit a poor prognosis (1–4). A previous study has reported the overall five-year survival rate following radical resection to be 15–50%, as cholangiocarcinoma cells easily metastasize, resulting in relapse (5). The formation of cholangiocarcinoma often proceeds through several steps, including interaction among the cancer-promoting environmental factors, oncogene activation and tumor-suppressor gene inactivation. The relative balance controlling these processes is broken following chronic injury to the cholangiocytes. During the tumorigenesis of cholangiocarcinoma, certain growth factors, receptors and signaling pathway molecules may be involved (1).

Raf kinase inhibitor protein (RKIP), also known as human phosphatidylethanolamine binding protein 1, is a soluble basic protein with a molecular weight of 21–23 kD and an isoelectric point of ~8.6. RKIP is a highly conserved small molecular protein that has been well preserved during evolution (6). RKIP is mainly located in the cytoplasmic organelles and on the plasma membrane. Previous studies have found that RKIP affects various cellular processes. The protein can inhibit MAP kinase (Raf-MEK-ERK), G protein-coupled receptor (GPCR) kinase (GRK) and nuclear factor-κB (NFκB) signaling cascades. Non-phosphorylated RKIP interferes with Raf-1 activity, disrupts the interaction between Raf and MEK, and prevents the activation of MEK and its downstream components, resulting in the negative regulation of the Raf-MEK-ERK pathway. By contrast, phosphorylated RKIP dissociates from Raf-1, but binds and inhibits G-protein coupled receptor kinase (GRK)-2, resulting in sustained G-protein signaling (6–8).

RKIP is implicated in several types of cell behavior, including cell growth, apoptosis, invasion and migration (8). It has previously been indicated that RKIP expression is decreased in metastatic prostate and breast cancer. Furthermore, the overexpression of RKIP in metastatic prostate and breast cancer can decrease the invasive ability of these cells. RKIP overexpression is induced in breast and prostate cancer cells when they are treated with chemotherapeutic agents, predisposing the cells to apoptosis (9). It has been proposed that RKIP expression can be used as a prognostic marker for renal cell carcinoma patients (10). Another study has found that RKIP can inhibit breast tumor metastasis through the enhancement of microRNA let-7 processing (11). Additionally, the level of RKIP expression is low in lung, bladder and cervical cancers and nasopharyngeal carcinoma (12). Our previous study found that RKIP may prevent liver fibrosis, which is a pre-cancerous lesion, through its inhibitory effects on hepatic stellate cell (HSC) proliferation (13). We have also previously demonstrated an association between lymph node or distant metastasis in decreased RKIP expression and esophageal cancer tissues. The study demonstrated that RKIP downregulates the expression of GRK-2, Lin28 and matrix metalloproteinase (MMP)-14 (14). However, the association between RKIP expression and the progression of cholangiocarcinoma, and the effect of its regulation on the biological behavior of cholangiocarcinoma cells is not yet clear. Therefore, the present study investigated the association between RKIP expression and the prognosis of cholangiocarcinoma, and the effects of the protein on cholangiocarcinoma cell growth, apoptosis, invasion and metastasis.

## Materials and methods

### Subjects

In total, 30 extrahepatic cholangiocarcinoma tumor and adjacent uninvolved peritumoral tissues were obtained at the time of surgery from patients in the Second Hospital of Hebei Medical University (Shijiazhuang, Hebei, China). The tissues were fixed in 10% neutral-buffered formalin overnight and were embedded in paraffin in a tissue processor. The present study was approved by the Ethics Committee of the Second Hospital of Hebei Medical University, in line with ethics requirements. Written informed consent was obtained from all patients.

### Immunohistochemical staining

The paraffin-embedded tissue specimens were sectioned to a 4-μm thickness and mounted on Adhesion Microscope slides (Citotest Labware Manufacturing Co., Ltd., Haimen, China). The tissue sections were deparaffinized, rehydrated and stained by hematoxylin and eosin (HE) or used for immunohistochemistry (IHC) with mouse monoclonal anti-human cytokeratin (CK)19 (1:1, Fuzhou Maixin Biotechnology Development Co., Ltd., Fuzhou, China) or rabbit anti-human polyclonal RKIP (1:200; Santa Cruz Biotechnology, Inc., Santa Cruz, CA, USA) antibodies.

The sections were stained and the RKIP staining results were scored by a previously described method (14,15). Tissues with a final score that exceeded the median score were classed as having high RKIP expression and tissues with a final score equal to or below the median exhibited downregulated RKIP expression. The correlations between RKIP expression in the cholangiocarcinoma tissue and age, gender, differentiation, pathological stage and lymph node or distant metastases were investigated.

### Design and cloning of short hairpin RNA constructs

Two 21-nucleotide (nt) small-interfering (si)RNA sequences of *Homo sapiens* (Genbank accession, NM_002567) were designed using the GenScript web-based program (http://www.genscript.com/siRNA_service.html, GenScript USA Inc., Piscataway, NJ, USA). The specificity of the siRNA sequences was verified by a Basic Local Alignment Search Tool search (http://blast.ncbi.nlm.nih.gov/Blast.cgi). The siRNA sequence was 5′-CGAGCAGCTGTCTGGGAAGTA-3′. A non-related 19-nt sequence, 5′-TTCTCCGAACGTGTCACGT-3′, was used as an siRNA-negative control. To employ viral delivery of the double-stranded siRNA, the adenoviral vector, pDC316-siRNA (Shanghai Genechem Co., Ltd., Shanghai, China), was used. The successful pDC316-siRNA recombinant vector [RKIP-RNA interference (RNAi)-AD] production was confirmed by sequencing. A negative siRNA control with green fluorescent protein (GFP; NC-RNAi-GFP-AD) was also used.

### Preparation of the RKIP-overexpressing vector

To induce RKIP overexpression *in vitro*, an adenoviral vector expressing RKIP (RKIP-AD; Genbank accession, NM_002567) and driven by a mCMV promoter was constructed. This was accomplished by placing the complementary (c)DNA for RKIP, excised by *Age*I from plasmid pDC315-enhanced GFP (EGFP; Shanghai Genechem Co., Ltd.), downstream of the mCMV gene promoter. Recombinant virus from a single plaque was identified by DNA analysis, expanded in NIH293 cells (American Type Culture Collection, Manassas, VA, USA), and twice purified by an Adeno-XTM Virus Purification kit (BD Biosciences, Clontech, San Jose, CA, USA). The viral titers were determined by median tissue culture infective dose (TCID_50_) assays. The titers determined by the TCID_50_ assays were used in subsequent experiments. No contamination was detected in the viral preparations used in the experiments. The adenoviral vector pDC315-EGFP (GFP-AD) was used as a negative control.

### Infection of the human cholangiocarcinoma RBE cell line

The human cholangiocarcinoma RBE cell line was provided by the Cell Bank of the Chinese Academy of Sciences (Shanghai, China). The RBE cells were cultured in RPMI-1640, supplemented with 10% heat-inactivated fetal bovine serum (FBS), 100 IU/ml penicillin, 100 μg/ml streptomycin (Biochrom KG, Berlin, Germany) and 4 mmol/l L-glutamine under a humidified atmosphere of 5% CO_2_ and 95% air at 37°C, and subcultured when 90% confluent. In total, 1×10^6^ RBE cells were infected for 48 h with pretreated viral particles (RKIP-RNAi-AD, NC-RNAi-GFP-AD, RKIP-AD and GFP-AD) at a multiplicity of infection (MOI) of 400. The RKIP protein and mRNA expression was determined by western blot analysis and reverse transcription-quantitative polymerase chain reaction (RT-qPCR), respectively.

### Western blot analysis

Subsequent to being rinsed three times with phosphate-buffered saline (PBS), the RBE cells were lysed with lysis buffer and extracted on ice (13). The samples were separated by sodium dodecyl sulfate polyacrylamide gel electrophoresis and electroblotted onto polyvinylidene fluoride membranes (Millipore, Bedford, MA, USA). The membranes were then incubated with primary antibodies at 4°C overnight. The primary antibodies were rabbit anti-human polyclonal RKIP (1:400) and rabbit anti-human polyclonal glyceraldehyde 3-phosphate dehydrogenase (GAPDH) (1:1,000; Santa Cruz Biotechnology, Inc.). Subsequent to being washed with Tris-buffered saline with Tween 20, the membranes were incubated with horseradish peroxidase-conjugated secondary antibodies (Santa Cruz Biotechnology, Inc.) for 2 h at room temperature. The antigens were detected by enhanced chemiluminescence (Santa Cruz Biotechnology, Inc.). For protein quantification, the bands were scanned and quantified by NIH ImageJ 1.38 software (National Institutes of Health, Bethesda, MD, USA), using GAPDH as the internal control. The results were reported as the mean of triplicate assays.

### RT-qPCR assay

The total RNA from the RBE cells was isolated using TRIzol reagent (Tiangen Biotech (Beijing) Co., Ltd., Beijing, China), according to the manufacturer’s instructions (14). Overall, ~200 ng of total RNA was converted to first-strand cDNA using a SuperScript-II RT system (Life Technologies, Carlsbad, CA, USA). qPCR was performed in a total volume of 20 μl in the presence of SYBR Green PCR master mix [Tiangen Biotech (Beijing) Co., Ltd] on an ABI Stepone Plus Real-Time PCR Systems device (Applied Biosystems, Foster City, CA, USA). The primers used to amplify RKIP, MMP-9, tissue inhibitor of metalloproteinase 4 (TIMP-4) and GAPDH were as follows: RKIP forward, 5′-AGACCCACCAGCATTTCGTG-3′ and reverse, 5′-GCTGATGTCATTGCCCTTCA-3′; MMP-9 forward, 5′-CACCGCCAACTACGACCGGG-3′ and reverse, 5′-GGTGGTAGCGCACCAGAGGC-3′; TIMP-4 forward, 5′-AAGAGCCTCGGGTCCTGCCTC-3′ and reverse, 5′-CAA GGCCGTTGTGCCCCTCG-3′; and GAPDH forward, 5′-GAACGGGAAGCTCACTGGCATGGC-3′ and reverse, 5′-TGAGGTCCACCACCCTGTTGCTG-3′. The PCR conditions were as follows: 10 min incubation phase at 95°C, 40 cycles of 95°C for 10 sec, 60°C for 30 sec, and 72°C for 20 sec. Results in triplicate were expressed as the ratio of the cycle threshold value for the target gene cDNA concentration relative to that for GAPDH, using the 2^−ΔΔCt^ method.

### Cell proliferation assay

RBE cells in the logarithmic growth phase were trypsinized and seeded in a 96-well plate, 200 μl (5×10^4^ cells/ml) per well, and incubated with RKIP-RNAi-AD, NC-RNAi-GFP-AD, RKIP-AD, GFP-AD or PBS for 24, 48, 72 or 96 h. Cell proliferation was evaluated with a 3-(4,5-dimethylthiazol-2-yl)-2,5-diphenyltetrazolium bromide (MTT) assay (Sigma-Aldrich, St. Louis, MO, USA). The absorbance was determined at a 492-nm wavelength, with a reference wavelength of 630 nm. The results are presented as the average absorbance of 6 wells in one experiment, and the assays were performed in triplicate.

### Apoptosis assay

The flow cytometry data of the RBE cells were collected following adenoviral-mediated transfer, centrifugation at 700 × g for 5 min and resuspension in PBS at a concentration of 5×10^5^ cells/ml. Annexin V-phycoerythrin was added and the cells were kept away from light for 15 min at room temperature until the analysis. The DNA content was analyzed using a flow cytometer (Coulter Epics XL; Beckman Coulter, Inc., Brea, CA, USA). Cell apoptosis was analyzed using the WinMDI software program (Scripps Research Institute, La Jolla, CA, USA).

### Transwell invasion assay

RBE cells (5×10^5^ per well) at 48 h post-transfection were resuspended in 200 μl serum-free medium containing 1% bovine serum albumin and were seeded on the top chamber of the 8-μm pore Transwell, using 6.5-mm polycarbonate Transwell filters (Corning, Inc., New York, NY, USA). The bottom chamber was supplied with 600 μl of medium containing 10% FBS. After a 48-h incubation period in CO_2_ at 37°C, the non-invading cells were removed from the upper surface of the membrane by gentle scrubbing with a cotton swab. The cells that had invaded the bottom chamber were fixed with methanol and stained with crystal violet solution. The number of cells on the lower surface of the filters was counted by two researchers blinded to the sample groups. A total of five representative fields were counted for each Transwell filter.

### Wound healing assay

RBE cells (1×10^6^ cells/well) at 12 h post-transfection were seeded in six-well plates and allowed to adhere for 36 h. Confluent monolayer cells were scratch wounded using a sterile plastic micropipette tip and then washed three times with PBS to clear any cell debris and suspended cells. Fresh serum-free medium was added and the cells were allowed to close the wound for 24 h. Images were captured at 0, 12 and 24 h at the same wound position. Morphometric analyses of the digital images were then performed with NIH ImageJ 1.38 software. The percentage of wound closure was revealed by the change in the area of the wound that remained open at each time-point. Curve fitting and statistical analyses were carried out by GraphPad Prism software (GraphPad Software, Inc., La Jolla, CA, USA).

### Statistical analysis

Statistical analyses were performed by SPSS 13.0 (SPSS, Inc., Chicago, IL, USA). The metrological data are presented as the mean ± standard deviation. The χ^2^, Kruskal-Wallis, Spearman’s rank correlation coefficient, one-way analysis of variance and Fisher’s least significant difference tests were used as appropriate. P<0.05 was considered to indicate a statistically significant difference.

## Results

### RKIP expression is decreased in cholangiocarcinoma tissues

**Figure 1:**
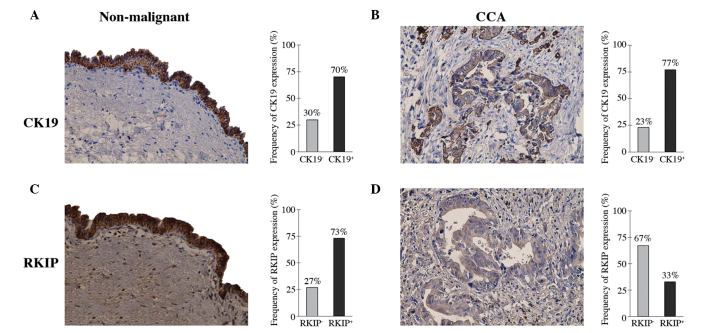
Extrahepatic cholangiocarcinoma tumors and adjacent uninvolved peritumoral tissues immunostained with anti-CK19 and anti-RKIP polyclonal antibodies. The positive staining for CK19 and RKIP protein is indicated by a reddish-brown color. Abundant yellowish-brown particle sediment was observed in the cytoplasm of the basal, parabasal and superficial cells. CK19 was constitutively expressed in (A) cholangiocytes (magnification ×400) and (B) cholangiocarcinoma cells (magnification ×400). (C) RKIP immunoreactivity was detected in adjacent uninvolved peritumoral tissues (magnification ×400) and (D) a lower frequency of RKIP expression was observed in cholangiocarcinoma tissues (magnification ×400). These results demonstrate that RKIP expression was significantly reduced in the cholangiocarcinoma tissues. RKIP, Raf kinase inhibitor protein; CK, cytokeratin; CCA, cholangiocarcinoma.

HE staining of the specimens obtained from 30 patients with cholangiocarcinoma revealed evident differences between the specimens of the extrahepatic cholangiocarcinoma tumor and adjacent uninvolved peritumoral tissues, for which representative images from single similar experiments are provided. CK19 was constitutively expressed in the normal cholangiocytes and cholangiocarcinoma cells and there was no significant difference between the two groups (χ^2^ test, P>0.05; Fig. 1A and B).

Immunohistochemical staining of the RKIP polyclonal antibody revealed high RKIP expression in the normal cholangiocytes, with abundant yellowish-brown particle sediment appearing in the cytoplasm. However, RKIP expression was lower in the cholangiocarcinoma cells, with no evident yellowish-brown particle sediment being observed. The frequency of RKIP-positive expression was 73 vs. 33% in the normal cholangiocytes and cholangiocarcinoma, respectively (Fig. 1C and D). These results indicated that the RKIP expression was significantly lower in the cholangiocarcinoma cells compared with the normal cholangiocytes (χ^2^ test; P<0.01).

### RKIP expression is associated with cell differentiation and the lymph node or distant metastasis of cholangiocarcinoma

The age of the cholangiocarcinoma patients, 12 male and 18 female, ranged between 45 and 75 years old. The 30 cholangiocarcinoma tumors were all identified as adenocarcinoma by HE staining. In total, 8 patients presented with well-differentiated cholangiocarcinoma, 9 with moderately-differentiated cholangiocarcinoma and 13 with poorly-differentiated cholangiocarcinoma. Of the 30 patients with regional lymph node involvement, 17 exhibited early-stage (I–II) disease and 13 exhibited advanced-stage (III–IV) disease. In addition, 13 patients presented with lymph node or distant metastases.

RKIP expression was negatively correlated with cell differentiation (P<0.001) and lymph node or distant metastasis (P<0.01). However, RKIP expression did not correlate with the age or gender of the patient or the pathological stage of the tumor (Table I).

### RKIP overexpression or downregulation in RBE cells infected by adenoviral vectors does not affect RBE cell proliferation and apoptosis

**Figure 2:**
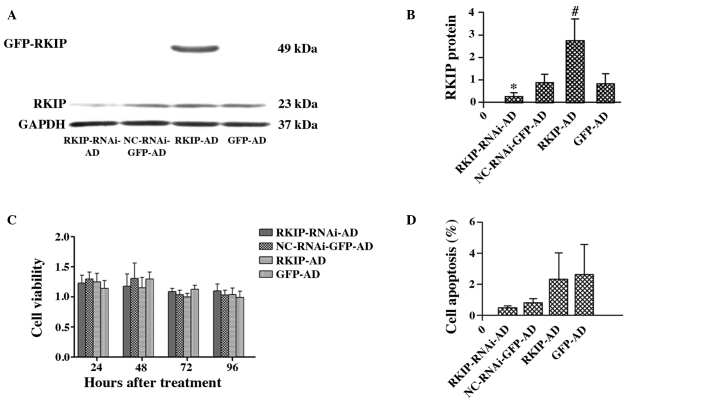
Western blot analysis to detect RKIP and GAPDH protein expression in RBE cells 48 h after adenoviral infection. (A and B) RKIP expression is decreased in the RKIP-RNAi-AD treatment group compared with the NC-RNAi-GFP-AD group (^*^P<0.05; n=3). In the RKIP-AD group, a large amount of RKIP is expressed as an exogenous protein compared with the GFP-AD group (^#^P<0.05; n=3). (C) Cell viability of the RBE cells exposed to recombinant adenovirus at different time-points, assessed by MTT assay. The RBE cells were transfected with recombinant adenovirus for 24, 48, 72 or 96 h, respectively. These cells were infected with RKIP-RNAi-AD, NC-RNAi-GFP-AD, RKIP-AD or GFP-AD. The differences were not significant in either of the two groups (P>0.05; n=3). (D) Apoptosis of the RBE cells at 48 h after recombinant adenovirus infection. Apoptosis of the RBE cells transfected with RKIP-RNAi-AD, NC-RNAi-GFP-AD, RKIP-AD and GFP-AD, respectively, was detected by phycoerythrin-labeled flow cytometry. The differences were not significant in either of the two groups (P>0.05; n=3). RKIP, Raf kinase inhibitor protein; RKIP-RNAi-AD, siRNA recombinant vector; NC-RNAi-GFP-AD, siRNA-negative control with green fluorescent protein (GFP); RKIP-AD, adenoviral vector expressing RKIP; GFP-AD, adenoviral negative control.

To further elucidate the role of RKIP, the RBE cells were infected with RKIP siRNA or RKIP-overexpressing adenoviral vector. The adenoviral infection efficiency in the different groups was first determined using flow cytometry. The infection rates of RKIP-RNAi-AD, NC-RNAi-GFP-AD, RKIP-AD and GFP-AD in the RBE cells were 87.9, 91.5, 94.5 and 89.4%, respectively, at 48 h post-transfection (data not shown). Next, western blot analysis was used to further confirm that the RKIP vector was transiently transfected into the RBE cells. The data revealed that RKIP expression in the RKIP-AD group was significantly increased compared with the GFP-AD group (2.77±0.95 vs. 0.85±0.44; P<0.05). Compared with the NC-RNAi-GFP-AD group, RKIP expression was significantly decreased in the RKIP-RNAi-AD group (0.26±0.17 vs. 0.88±0.37; P<0.05; Fig. 2A and B).

Adenovirus-mediated gene transfection at an MOI of 400 was used for the subsequent experiments. MTT assays revealed that RKIP-RNAi-AD or RKIP-AD adenoviral infection for 24, 48, 72 and 96 h had no effect on the viability of the RBE cells (P>0.05; Fig. 2C). Flow cytometry demonstrated that there was no significant difference in the apoptotic rate of the RBE cells between the RKIP-RNAi-AD (0.47±0.15%) and NC-RNAi-GFP-AD groups (0.80±0.26%) (P>0.05). Similarly, there was no significant difference in the apoptotic rate of the RBE cells between the RKIP-AD (2.3±1.71%) and GFP-AD groups (2.6±1.96%) (P>0.05; Fig. 2D).

### RKIP inhibits RBE cell invasion

**Figure 3:**
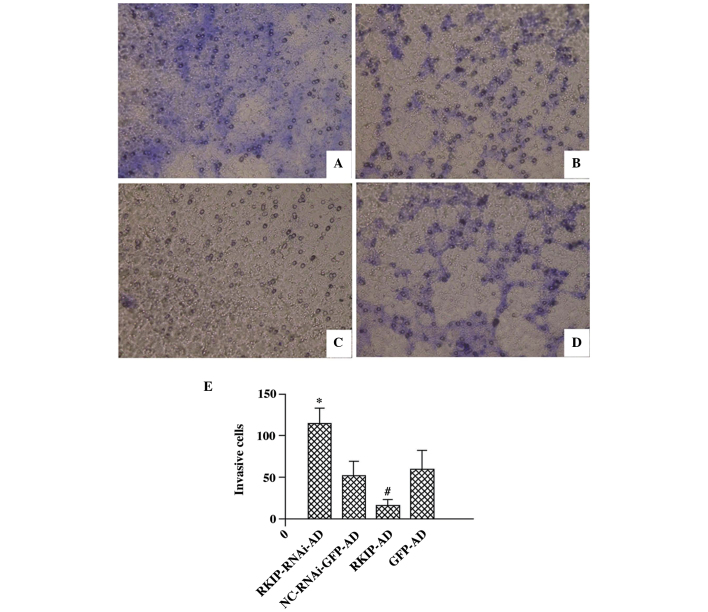
Invasive ability of RBE cells 48 h after recombinant adenoviral infection, as detected by invasion assay (magnification, ×200). The RBE cells were infected with (A) RKIP-RNAi-AD, (B) NC-RNAi-GFP-AD, (C) RKIP-AD and (D) GFP-AD, respectively. (E) Number of cells on the lower Transwell filter for each condition, revealing that RKIP inhibits the invasion of RBE cells (^*^P<0.05 vs. NC-RNAi-GFP-AD; ^#^P<0.05 vs. GFP-AD; n=6 for each group). RKIP, Raf kinase inhibitor protein; RKIP-RNAi-AD, siRNA recombinant vector; NC-RNAi-GFP-AD, siRNA-negative control with green fluorescent protein (GFP); RKIP-AD, adenoviral vector expressing RKIP; GFP-AD, adenoviral negative control; RNAi, RNA interference.

Next, the effect of RKIP on the invasive ability of the RBE cells was investigated (Fig. 3A–E). There was an increase in the number of invasive cells in the RKIP-RNAi group compared with the NC-RNAi-GFP-AD group (115.00±18.30 vs. 52.67±16.62; P<0.05), while there was a decrease in the number of invasive cells in the RKIP-AD group compared with the GFP-AD group (17.00±6.48 vs. 60.67±22.02; P<0.05). However, the cell invasion assay revealed that there was no significant difference between the GFP-AD and NC-RNAi-GFP-AD groups (60.67±22.02 vs. 52.67±16.621; P>0.05; Fig. 3E).

### RKIP inhibits RBE cell migration

**Figure 4:**
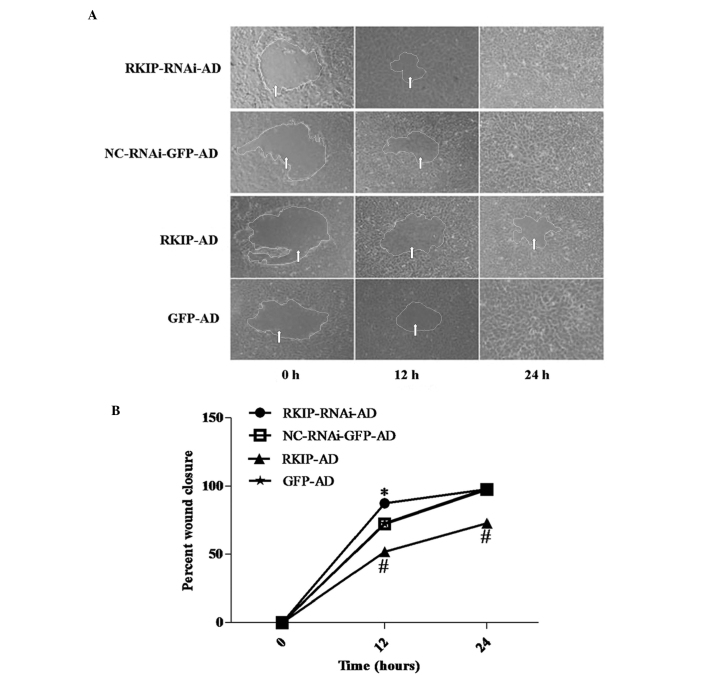
Wound closure assay. At 12 h, RKIP RNAi promoted wound closure, while RKIP-overexpression inhibited wound closure. (A) A wound was created in confluent RBE cell monolayers and the percentage of wound closure was revealed by the change in the area of the wound that remained open at each time-point, and (B) these values were plotted. RKIP RNAi enhanced wound closure (^*^P<0.05 vs. NC-RNAi-GFP-AD; n=6 for each group); RKIP overexpression inhibited wound closure in the RBE cells (^#^P<0.01 vs. GFP-AD; n=6 for each group). RKIP, Raf kinase inhibitor protein; RKIP-RNAi-AD, siRNA recombinant vector; NC-RNAi-GFP-AD, siRNA-negative control with green fluorescent protein (GFP); RKIP-AD, adenoviral vector expressing RKIP; GFP-AD, adenoviral negative control; RNAi, RNA interference. Arrows indicate the extent of the wound.

The wound closure assay revealed that at 12 h, the RBE cells in the RKIP-RNAi-AD group grew to close 87.6% of the wound, while the cells in the NC-RNAi-GFP-AD group only closed 72.3%; the difference between the two groups was statistically significant (P<0.05). RKIP overexpression significantly inhibited the wound closure rate of the RBE cells in the RKIP-AD group, with only 52.2% of the wound being closed, while 72.8% was closed in the GFP-AD group (P<0.05; Fig. 4). At 24 h, there was no significant difference between the RKIP-RNAi-AD and NC-RNAi-GFP-AD groups. However, compared with GFP-AD, RKIP-AD significantly inhibited RBE cell migration (98.4 vs. 73.1%; P<0.01; Fig. 4B).

### RKIP downregulates MMP-9, but upregulates TIMP-4 mRNA expression

**Figure 5:**
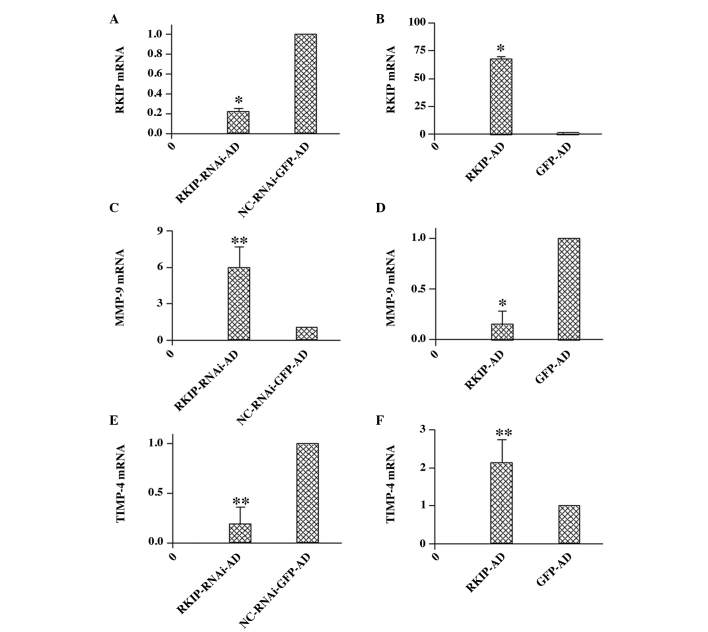
Effect of RKIP RNAi and overexpression on the mRNA expression of RKIP, MMP-9 and TIMP-4 in RBE cells. RKIP, MMP-9 and TIMP-4 mRNA expression was assessed by quantitative polymerase chain reaction. The expression was normalized as a ratio using GADPH as a reference gene. A value of 1 for this ratio was arbitrarily assigned to the data obtained from the control. (A) RKIP mRNA expression was significantly suppressed in the RKIP-RNAi-AD group in RBE cells (^*^P<0.01) and (B) RKIP mRNA expression was significantly increased in the RKIP-AD group in the RBE cells (^*^P<0.01). (C) The expression of the MMP-9 transcripts was increased by RKIP RNAi (^**^P<0.05), (D) and the expression of the MMP-9 transcripts was significantly reduced in the RKIP-AD group (^*^P<0.01). (E) The expression of the TIMP-4 transcripts was reduced by RKIP RNAi (^**^P<0.05) and (F) the expression of TIMP-4 transcripts was significantly increased in the RKIP-AD group (^**^P<0.05). All the comparisons in (A), (C) and (E) were made between RBE cells transfected with RKIP-RNAi-AD and those with the vector, NC-RNAi-GFP-AD. All the comparisons in (B), (D) and (F) were made between RBE cells transfected with RKIP-AD and those with the vector GFP-AD. All the data are expressed as the mean ± standard deviation from three individual experiments (n=3). RNAi, RNA interference; RKIP, Raf kinase inhibitor protein; MMP, matrix metalloproteinase; TIMP, tissue inhibitor of metalloproteinase; RKIP-RNAi-AD, siRNA recombinant vector; NC-RNAi-GFP-AD, siRNA-negative control with green fluorescent protein (GFP); RKIP-AD, adenoviral vector expressing RKIP; GFP-AD, adenoviral negative control.

Following the silencing or overexpression of RKIP by RKIP siRNA or adenoviral vectors, respectively, RT-qPCR was performed to analyze the mRNA expression levels of RKIP, MMP-9 and TIMP-4, using a quantitative method. When RKIP-RNAi-AD (0.22±0.04) was compared with NC-RNAi-GFP-AD (1.00±0.00), a significant decrease in RKIP mRNA expression levels was revealed (P<0.01, Fig. 5A). However, RKIP mRNA expression was significantly higher in the RKIP-AD group (67.22±2.49) compared with the GFP-AD group (1.00±0.00; P<0.01) (Fig. 5B).

The mRNA expression of MMP-9 was significantly higher in the RKIP-RNAi-AD group (5.96±1.70) compared with the NC-RKIP-RNAi-AD group (1.00±0.00) (P<0.05; Fig. 5C). However, the MMP-9 mRNA expression was significantly lower in the RKIP-AD group (0.15±0.13) compared with the GFP-AD group (1.00±0.00) (P<0.01; Fig. 5D). In addition, the mRNA expression of TIMP-4 was significantly lower in the RKIP-RNAi-AD group (0.19±0.17) compared with the NC-RKIP-RNAi-AD group (1.00±0.00) (P<0.05; Fig. 5E), and higher in the RKIP-AD group (2.13±0.60) compared with the GFP-AD group (1.00±0.00) (P<0.05; Fig. 5F).

## Discussion

The current study provides evidence that RKIP expression is decreased in cholangiocarcinoma tissues, and that it negatively correlates with cholangiocarcinoma cell differentiation and lymph node or distant metastasis. In the present study, RKIP was revealed to inhibit the invasion and metastasis of cholangiocarcinoma cells by downregulating MMP-9, but upregulating TIMP-4 mRNA expression.

Cholangiocarcinoma are rare, malignant tumors that originate in the biliary tract epithelia. Based on their anatomical location, cholangiocarcinomas are classified as either intrahepatic or ductal cholangiocarcinoma. Liver fluke infestations, chronic viral hepatitis, hepatolithiasis, choledochal cysts and primary sclerosing cholangitis can all predispose patients to developing cholangiocarcinoma (16). The tumor-node-metastasis classification for biliary tract cancers is not useful in clinical practice, as the T classification does not differentiate between the prognosis, for example, of T2 and T3 tumors. Surgery is the main treatment for resectable, localized cholangiocarcinoma. However, it is extremely easy for cholangiocarcinoma cells to metastasize and cause relapse. The number of lesions and the presence of vascular invasion have been found to be important prognostic factors, whereas tumor size has not (17,18).

RKIP is a multifaceted kinase modulator that is conserved between species. RKIP inhibits the Raf-MEK-ERK, GPCR kinase and NFκB signaling cascades. RKIP can be phosphorylated at serine 153 following protein kinase C stimulation. Residues 127–146 of RKIP are critical for dimer formation. The formation of the dimer is an important mechanical feature in the switch in RKIP association from Raf1 to GRK2 (19–21). Several studies have demonstrated that RKIP is closely associated with numerous tumors and participates in their occurrence and development. RKIP expression is decreased in numerous tumors and regulates the growth, apoptosis, invasion and metastasis of tumor cells (8–12,14).

CK19 is a highly sensitive cholangiocyte marker and is also commonly overexpressed in cholangiocarcinoma cells (22). In the present study, CK19, as a specific diagnostic marker, was stained in the extrahepatic cholangiocarcinoma tumor and adjacent uninvolved peritumoral tissues. Immunohistochemical staining revealed that RKIP expression was decreased in cholangiocarcinoma tissues, which is similar to the findings of a previous study, which revealed that RKIP expression contributes to invasion and metastasis in carcinoma of the ampulla of Vater (23). The current study further demonstrated that reduced RKIP expression in cholangiocarcinoma cells is negatively correlated with cell differentiation and lymph node or distant metastasis. Therefore, positive RKIP expression in cholangiocarcinoma cells may indicate a good prognosis for patients.

To further explore the role of RKIP in cholangiocarcinoma growth, in the present study, the protein was either overexpressed through an RKIP adenoviral vector or silenced by RKIP siRNA. It was found that neither overexpressed nor downregulated RKIP affected cholangiocarcinoma cell proliferation *in vitro*. At the same time, neither RKIP overexpression nor RKIP-knockdown enhanced cell apoptosis.

RKIP has been identified as a member of a novel class of metastasis suppressors, with evidence from prostate cancer, breast cancer, malignant melanoma, insulinoma, colorectal cancer, hepatocellular carcinoma and esophageal cancer (14,24). However, the role of RKIP in cholangiocarcinoma metastasis requires elucidation. Immunostaining for RKIP in the present study indicated that reduced RKIP expression promotes cholangiocarcinoma cell metastasis. In order to evaluate the effect of RKIP on cholangiocarcinoma metastasis, the cholangiocarcinoma cell line, RBE, was infected by RKIP-overexpressing vectors *in vitro*. Cell invasion and wound closure assays revealed that RKIP not only inhibits RBE cell invasion, but that it also suppresses cell migration. By contrast, the RKIP-targeting siRNA vector promoted RBE cell invasion and cell migration. A lack of RKIP expression can make the cholangiocarcinoma cells susceptible to invasion and migration.

Several prognostic factors are involved in the evaluation of cholangiocarcinoma. Among these, lymph node and distant metastasis is one of the most important prognostic factors (25–27). Metastasis is a multi-step process that involves the spread of cancer cells from the primary site to a secondary location. During this process, the cancer cells must invade the surrounding tissue, penetrate the blood or lymphatic vessels and form a novel tumor mass at a distant site. During invasion, the cancer cells produce MMPs and then degrade the extracellular matrix (ECM) and basement membrane to generate space for the cells to migrate out of the original site. The TIMPs play an important role in maintaining the balance between ECM synthesis and the degradation caused by MMPs. TIMPs are usually downregulated in cancer cells. In addition, it has been determined that MMPs-2, -7 and -9 and TIMPs-1, -2 and -3 are candidate cancer-promoting or suppressor genes in the progression of cholangiocarcinoma (28–32). RKIP has been revealed to prevent the invasion of cancer cells by controlling the gene expression of MMPs, particularly MMP-1 and MMP-2 (12). RKIP also inhibits esophageal cancer cell invasion via the downregulation of MMP-14 expression (13). The present study demonstrated that RKIP overexpression significantly inhibits MMP-9 expression, indicating that RKIP reduces the invasiveness of cholangiocarcinoma RBE cells by downregulating MMP-9 expression. In addition, RKIP significantly enhanced TIMP-4 gene expression, playing a complementary role in suppressing cholangiocarcinoma cell invasion.

In conclusion, the present results suggested that reduced RKIP expression is associated with cholangiocarcinoma metastasis. Positive RKIP expression in cholangiocarcinoma cells may be be predictive of a better prognosis. RKIP inhibits cholangiocarcinoma cell invasion and migration via downregulating MMP-9 expression and upregulating TIMP-4 expression.

## Figures and Tables

**Table I:** Correlations between clinicopathological characteristics and RKIP expression.

		RKIP expression		
			
Parameters	Total (n=30)	High	Low	P-value
Mean age ± SD, years	59±10	58±8	60±12	0.451^a^
Gender, n				0.654^b^
Male	12	7	5	
Female	18	9	9	
Differentiation, n				<0.001^b^
Good	8	8	0	
Moderate	9	6	3	
Poor	13	2	11	
pStage, n				0.961^b^
I–II	17	9	8	
III–IV	13	7	6	
Lymph or distant metastasis, n				0.004^b^
Yes	13	3	10	
No	17	13	4	

a Student’s t-test.

b χ^2^ test.

RKIP, Raf kinase inhibitor protein; SD, standard deviation; pStage, pathological stage.
